# Effect and Mechanism of Endothelin Receptor A Inhibitor BQ-123 Combined with Electroacupuncture on Tibia Cancer Pain in Rats

**DOI:** 10.1155/2022/8563202

**Published:** 2022-05-17

**Authors:** Jie Tang, Zhilu Sun, Yan Wang, Jing Liu, Shuai Wang, Xilian Wang

**Affiliations:** ^1^The First Affiliated Hospital, Department of Pain, Hengyang Medical School, University of South China, 421001, China; ^2^The First Affiliated Hospital, Department of Emergency, Hengyang Medical School, University of South China, 421001, China; ^3^The First Affiliated Hospital, Department of Pathology, Hengyang Medical School, University of South China, 421001, China; ^4^The First Affiliated Hospital, Department of Rehabilitation, Hengyang Medical School, University of South China, 421001, China; ^5^The First Affiliated Hospital, Department of Hand Foot Surgery, Hengyang Medical School, University of South China, 421001, China

## Abstract

**Objective:**

To research the impact and mechanism of endothelin receptor A inhibitor BQ-123 combined with electroacupuncture on tibia cancer pain in rats.

**Methods:**

Sprague-Dawley (SD) rats were randomly divided into sham group (SHAM group) and bone cancer pain model group (BCP group). The behavior of SD rats was measured. The histology of the right tibia was observed by hematoxylin-eosin (HE) staining. The remaining rats were randomly divided into model, BQ-123, electroacupuncture, and BQ-123+ electroacupuncture group. Behavioral tests were performed, and mechanical pain threshold (MWT) and thermal pain threshold (TWL) were measured. The expressions of *α*-smooth muscle actin (*α*SMA), ETAR (endothelin A receptor), ETB (End of Transmission Block), P-Phosphatidylinositol 3-kinase (PI3K), and P-Protein kinase B (Akt) were detected by real-time fluorescence quantitative PCR and western blot.

**Results:**

In the BCP group, bone structure was severely damaged, local tissue swelling was obvious, bone trabecula was missing, and bone cortex was discontinuous. The optical density of Glial fibrillary acidic protein (GFAP) and CD11b immunoreactive signal in BCP group was significantly increased, and most of the ETAR of endothelin receptor was comapped with NeuN, and a small part of GFAP was comapped with CD11b, but no comapped with CD11b. The AS score of BQ-123+ electroacupuncture group was significantly lower than that of BQ-123 group and electroacupuncture group (*P* < 0.05), whereas the MWT and TWL values were significantly higher than that of the BQ-123 group and electroacupuncture group (*P* < 0.05). The mRNA expression of *α*-SMA and ETAR in BQ-123+ electroacupuncture group was lower than that in BQ-123 and electroacupuncture group, and the protein expression of P-PI3K and P-Akt in BQ-123+ electroacupuncture group was lower as well.

**Conclusion:**

BQ-123 may inhibit the activation of PI3K/Akt signal path combined with electroacupuncture to alleviate the effects of tibia cancer pain in rats.

## 1. Introduction

Bone cancer hurt is a vital issue for patients with advanced cancer because the pain induced by cancer seriously impacts the living quality of cancer patients. In cancer, the incidence of bone cancer hurt is high. According to statistics, the first feature of about 20-50% of cancerous persons is pain [1], and 70-90% of patients with terminal or end-stage cancer have chronic pain symptoms caused by incomplete treatment or cancer progression [2]. Hyperalgesia or hyperalgesia is an important indication for patients with cancer pain [3]. Hyperalgesia refers to pain stimulation caused by normal physiological state and can produce intense pain [4]. Hyperalgesia refers to pain response caused by pain stimulation caused by normal physiological state [5]. In the process of pain metastasis, spinal dorsal root ganglion can secrete a variety of cytokines, which can bind with corresponding receptors in tumor tissues and activate peripheral receptors, and the signals generated are transmitted into spinal dorsal horn through dorsal root ganglion and fibers, resulting in pain [6]. At present, analgesia is mainly treated with drugs. Clinical analgesics are generally divided into nonsteroidal anti-inflammatory drugs and opioid painkillers. These drugs can effectively relieve pain, but at the same time, many adverse reactions, such as nausea, vomiting, and gastrointestinal symptoms, will also produce strong dependence [7]. Therefore, it is of positive significance to choose the treatment with rapid effect and small side effects.

BQ-123 (cyclo[D-Asp-L-Pro-D-Val-L-Leu-D-Trp]) is an ETA-receptor antagonist, with an IC50 in binding experiments, of 7.3 nM for ETA-receptors, in porcine aortic smooth muscle cells, and a 2,500-fold selectivity for ETA relative to ETB-receptors in porcine cerebellum [8]. BQ-123 is an inhibitor of endothelin receptor A, and lots of research have indicated that BQ-123 has A protection effect on brain, heart, lung, and other important organs as well as diabetes [9]. However, the role of BQ-123 in bone cancer pain remains unknown. Therefore, this study is the first study for exploring the effects and mechanism of BQ-123 and electroacupuncture alone or in combination on tibia cancer pain in rats, providing new ideas for finding new drugs to treat cancer hurt.

## 2. Materials and Methods

### 2.1. Cell Culture

Walker 256 breast cancer cells were provided by the Shanghai Institute of Cancer Research, Chinese Academy of Science, and were added with 1640 culture medium of 10% serum 5 ml, mixed, centrifuged at 3000 rpm for 5 min, discarded supernatant, took 3 ml culture medium, blew cell suspension, added into culture flask, and placed in 37°C. Culture in 5% CO_2_ cell incubator. Culture to logarithmic growth phase, discard the supernatant, add 1640 cultured medium to mix thoroughly, continue culture, digestion and passage, and cell count, then centrifuge to remove the supernatant, clean with sterile PBS solution, use PBS solution to adjust the cell count of Walker 256 cells to 2 × 10^7^ cells/ml, and put in ice box for standby.

### 2.2. Modeling and Management of Tibial Cancer

Referring to the method for making a rat model of tibial cancer hurt [10], healthy female standard deviation rats were injected intraperitoneally with 10% C_2_H_3_Cl_3_O_2_ at 4 ml/kg body weight. The left hind limb was sterilized using 75% alcohol, and a blade was used to cut a small incision of about 1 cm at the most prominent bony position of the upper tibia of the left hind limb; then, fix both sides of the incision, bluntly separate the fascia and the periosteum on the surface of the tibia, and watch the lesser trochanter on the upper tibia. Punch a hole, insert the needle at a 45-degree angle about 5 mm below the tibial tubercle, then pull it out, and slowly inject 15 *μ*l of tumor cells into the cavum medullare of the upper tibia of the left hind limb with a microinjector, and leave it for 1 min after injection to prevent leakage. Bone wax was used to seal the marrow cavity, antibiotics were used, and sutures were sutured layer by layer.

### 2.3. Animal Grouping and Treatment

Thirty-four SD rats were randomly grouped into sham operation part (SHAM group) and bone cancer pain model part (BCP group) with 17 rats in each part. In the sham part, equal-volume heat-killed Walker 256 breast cancer cell suspension was injected into the left tibial bone marrow cavity during model establishment, and other operations remained unchanged. The BCP group was the rats modeled as described above. The day of modeling was 0 day, and behavioral tests were performed on 0 day, 1 day, 3 days, 6 days, 12 days, 15 days, and 18 days. On the 18th day, histological conditions of the right tibia of rats were observed by HE staining, and imaging examinations were conducted. Meanwhile, the distribution of ETAR in the spinal cord dorsal horn was tested by the immunofluorescence double-label method. Then, the remaining 76 rats were randomly grouped into 4 parts, including the model part, BQ-123 part, electroacupuncture part, and BQ-123+ electroacupuncture part, with 19 rats in each part. After BCP modeling, the model group was injected with intrathecal saline. After BCP modeling, the BQ-123 group was injected with 1 nmol/l from day 7 until day 18. On the 7th day after BCP modeling, electroacupuncture was given to the electroacupuncture group. Acupuncture was inserted into “Zusanli” and “Kunlun” points on the left side of the rats, and “density wave” was given through Han's acupoint nerve stimulator with the frequency of 100 Hz or 200 Hz, so as to cause slight jitter of the hind limb muscles of the rats. The treatment was performed once every 2 days, 30 min each time. Until the 18th day, after BCP modeling, the BQ-123+ electroacupuncture group was treated with BQ-123 injection and electroacupuncture, using the same method as above.

### 2.4. Walking Pain Score (AS) Was Determined

Regarding Kawakami's method [11] for quantitative scoring, the day of modeling is 0 day, and the sham group was evaluated at 9:00 am on the initial day, the first day, the third day, the sixth day, the twelfth day, the fifteenth day, and the eighteenth day. The AS was measured with the BCP group, and the walking observation box was self-made. The rats walked freely on the bottom of the box without any obstacles. After 10 minutes of adaptation, the walking pain score was measured. Normal walking and freedom of movement were scored as 0, mild claudication of the affected limb 2 points, moderate claudication of affected limb 3 points, and 4 points for severe lameness in the affected limb. The experiment was triplicate, once every 5 min, and the mean value was measured. Then, the rats in the model part, BQ-123 group, electroacupuncture group, and BQ-123+ electroacupuncture group were measured. The treatment began on the day of treatment, and the treatment was performed on the seventh day, the ninth day, the eleventh day, the thirteenth day, the fifteenth day, and the eighteenth day, respectively. AS was measured at 9:00 in the morning, as described above.

### 2.5. Determination of Mechanical Pain Threshold (MWT)

The up-and-down method [12] was used for the determination. In a quiet environment at room temperature of 21-23°C, the rats were put in a plexiglass case with a grid bottom. 0.6 g, 1 g, 1.4 g, 2 g, 4 g, 6 g, 8 g, 10 g, and 15 g of Von Frey pain cilia stimulate the paw of the rat in a certain order to observe the foot withdrawal response. The stimulation time of each Von Frey pain cilia was 2 s. The paw withdrawal reaction produced by the rat is positive. The Von Frey cilia with a lower pressure level will be used next time when a positive reaction occurs. After the first positive reaction, the test was performed 5 times, and the 50% mechanical foot withdrawal threshold was finally calculated. The calculation formula was 50% threshold = (10^[Xf + K*δ*]^)/10000. Xf is the logarithmic value of the cilia strength of the last stimulation, *K* is the coefficient of different stimulation methods, which can be obtained by looking up the table, and *δ* is the mean difference between the stimulations.

### 2.6. Determination of Heat Pain Threshold (TWL)

Thermal hyperalgesia was measured using a radiant thermal pain meter. In a quiet environment at room temperature of 21-23°C, the rats were placed in a plexiglass grid with a smooth bottom. After the rats were adapted for 20 minutes, the laser beam was irradiated on a large. In the center of the rat's paw, the rat's paw withdrawal latency was the time of the rat's paw withdrawal response, and the upper line of the rat's paw withdrawal latency was set to 20 s. The incubation period of claw contraction in rats was about 11 s, and each interval was 10 min. The mean value of the measurement was taken.

### 2.7. HE Dyed

On the 18th day after the operation, 2 rats in each group were taken and decapitated after anesthesia with chloral hydrate, the tibia on the inoculated side was taken, the soft tissue was stripped, and the tissue blocks were taken, fixed with 4% paraformaldehyde, embedded in paraffin, and cut. Bake slices into 4 µm slices at 60°C, place the slices in xylene for 2 times for 10 min each, wash with ethanol gradient for 5 min, stain with hematoxylin for 5 s, rinse back to blue, stain with eosin for 2 s, rinse and dehydrate with gradient alcohol, place in xylene for 10 min, seal with neutral gum, and observe under a microscope.

### 2.8. Imaging and Anatomic Observations

On the 18th day after the operation, sodium pentobarbital was shot into the abdominal cavity to anesthetize the rats. After the anesthesia was complete, a 50KVP intensity X-ray was used to take pictures of the affected limb to observe the changes in tibia structure. On the 18th day after surgery, the rats were anesthetized by intraperitoneal injection of C_2_H_3_Cl_3_O_2_. The tibia was dissected and the infiltration of the bone marrow cavity and the destruction of bone by tumor growth were observed.

### 2.9. Immunofluorescence Double Labeling Combined with Laser Confocal Fluorescence Microscopy Was Observed

The rats in each group were decapitated under deep anesthesia, and the spinal cord and lumbar dilation parts of the rats were rinsed in PBS and incubated for 2 h in blocking liquid containing sheep serum, BSA (Corning, NY), and 10%TritonX-100. Rabbit anti-mouse ETAR monoclonal antibody (1 : 400, Abcam, USA) was incubated overnight at 37°C, and the immunoflash double-standard experiment was performed. Mixed primary antibody was added. Mouse anti-human NeuN antibody (1 : 400, Abcam, USA), mouse anti-human glial fibrillary acidic protein antibody (1 : 1000), and mouse anti-HUMAN CD11b antibody (1 : 1000, Abcam, USA) were mixed with anti-mouse ETAR monoclonal antibody (1 : 400, Abcam, USA), placed overnight in the refrigerator at 4°C, rewarmed, and washed with PBS. Sheep anti-mouse IgG (1 : 1000, Abcam, USA) labeled with Alexa fluorescein 488 and 594 were incubated for 1 h in darkness, washed with PBS, sealed with antifluorescence quenching tablets, observed, and photographed by laser confocal fluorescence microscope.

### 2.10. Real-Time Fluorescence Quantitative PCR Detection

The rats in each part were given up under deep anesthesia, and the spinal cord and lumbar enlargement parts of the rats were segregated and put into EP tubes. The tissues were taken out and ground, and total RNA was drawn by Trizol method. The RNA content was determined by ultra-micro ultraviolet spectrophotometer, and cDNA was compounded by reverse transcription and detected by real-time fluorescence quantitative PCR. Using the principle of chimera fluorescent dye method, GoTaq® qPCR Master Mix kit was used for detection. PCR reaction conditions were 95°C, 2 min, 1 cycle, denaturation 95°C, 15 min, 40 cycles, annealing, and extension 60°C for 1 min. 2^-△△Ct^ was used to indicate the expression of target gene relative to internal reference GAPDH. Target gene expressions were calculated using the 2^−*ΔΔ*Ct^ method.

The primers were as follows:

- *α*-SMA upstream 5′-GGGTCACTCCCGTGTCAAAGT-3′ and downstream 5′-GTATcatgcatgCGACacag-3′
- ETAR upstream 5′-TGACTACTGTGGCGTCA-3′ and downstream 5′-GagtgCATatCGACGTTC-3′
- ETBR 5′-AGTCAGatAGTAAGAGT-3′ and downstream 5′-ACTCAGTGGCAGACGAAGG-3′
- Glyceraldehyde-3-phosphate dehydrogenase upstream 5′-CGGTCAAGTTCAAAGGcacA-3′ and downstream 5′-AGTGGCCATTAGCCTCCACG-3′

### 2.11. Western Blotting of Proteins

The rats in each part were given up under deep anesthesia, and the spinal cord and lumbar dilation parts of the rats were separated and placed into EP tubes. The tissues were ground, RIPA protein lysate was added, and protein was collected. The protein concentration of each group was measured by the BCA method. PI3K, AKT, p-PI3K, and P-Akt primary antibodies (Abcam, USA) were diluted at a ratio of 1 : 500, and GAPDH primary antibodies were diluted at 1 : 1000, incubated at 4°C overnight, thoroughly washed in TBST, diluted with goat anti-rabbit secondary antibody at 1 : 5000, incubated at room temperature for 2 h, took out PVDF membrane, washed with TBST, added ECL fluorescence detection reagent to PVDF membrane, pressed with X-ray film, developed in developing solution, and photographed with chemiluminescence gel image system.

### 2.12. Statistical Analysis

GraphPad Prism 9 software was used for data processing. Data with normal distribution were expressed as mean ± standard deviation$\left(\bar{x}\pm s\right)$. *T*-test was for pair-to-group comparison, and ANOVA was for multigroup comparison. The difference of *P* < 0.05 was statistically significant.

## 3. Results

### 3.1. Behavior and Bone Pain Were Measured in Rats after Modeling

**Figure 1:**
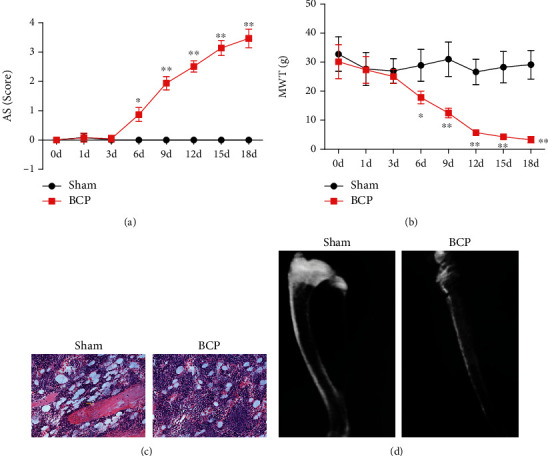
Behavior and bone pain in sham operation and model rats. (a) Walking pain score (AS). (b) Mechanical pain threshold (MWT) value. (c) HE staining picture (×200). (d) Imaging images of the affected limb.

Tibial HE staining results showed that on day 18 of the BCP part, lots of stained binucleated tumor cells were seen in the cavum medullare, and the bone structure was severely damaged. Normal bone marrow cells and bone trabecula were observed in the bone marrow cavity in the sham part. The tibia imaging examination showed that the BCP group rats showed obvious local tissue swelling, trabecular bone loss, and discontinuity of bone cortex. Three days before modeling, there was no statistically significant difference in AS score and MWT between the BCP group and sham part (*P* > 0.05); after 3 days, AS score of the BCP part was prominently higher than that of the sham part, the distinction was statistically significant (*P* < 0.05), MWT of BCP group was significantly lower than that of the sham group, and the difference was statistically significant (*P* < 0.05); AS is shown in Figure 1.

### 3.2. Distribution of ETAR in the Spinal Dorsal Horn

**Figure 2:**
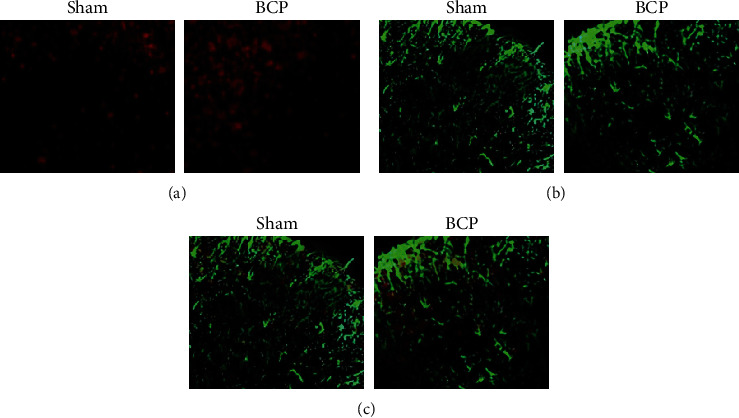
Immunofluorescence showed the distribution of ETAR in the spinal dorsal horn. (a) ETAR has a large number of common markers with neuron marker NeuN. (b) ETAR was colabeled with a small amount of astrocyte marker GFAP. (c) There was no costandard between ETAR and microglial cell marker CD11b.

Immunofluorescence results showed that contrasted with the sham part, the optical density of positive immunoreaction signals of NeuN, GFAP, and CD11b was prominently added in the BCP part, and most of the ETAR of the endothelin receptor was comapped with NeuN, and a small part of GFAP was comapped with CD11b, but no comapped with CD11b, as shown in Figure 2.

### 3.3. Effects of BQ-123 Combined with Electroacupuncture on Behavior of Rats in Different Time Groups

**Figure 3:**
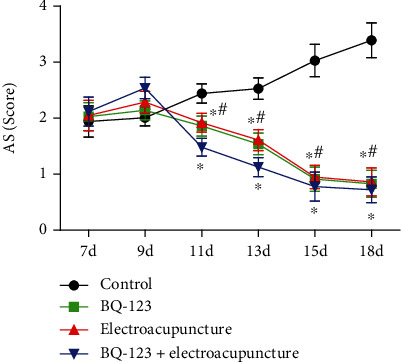
Effect of BQ-123 combined with electroacupuncture on the behavior of rats in different time groups.

We next assess the effects of BQ-123 combined with electroacupuncture on behavior of rats in different time groups. AS is shown in Figure 3; there was no statistical significance in the comparison of AS scores among groups at 7-9 days (*P* > 0.05). However, at 11-18 days, the AS score in the BQ-123 group and electroacupuncture part was significantly lower than that in the control group, and the distinction was statistically significant (*P* < 0.05), whereas the AS score of BQ-123 and electroacupuncture group existed no significant difference (*P* > 0.05). Furthermore, the score of BQ-123+ electroacupuncture part was lower than that of BQ-123 part and electroacupuncture group, and the distinction was statistically significant (*P* < 0.05).

### 3.4. Effects of BQ-123 Combined with Electroacupuncture on Anatomical and Histopathological Changes of the Tibia in Rats

**Figure 4:**
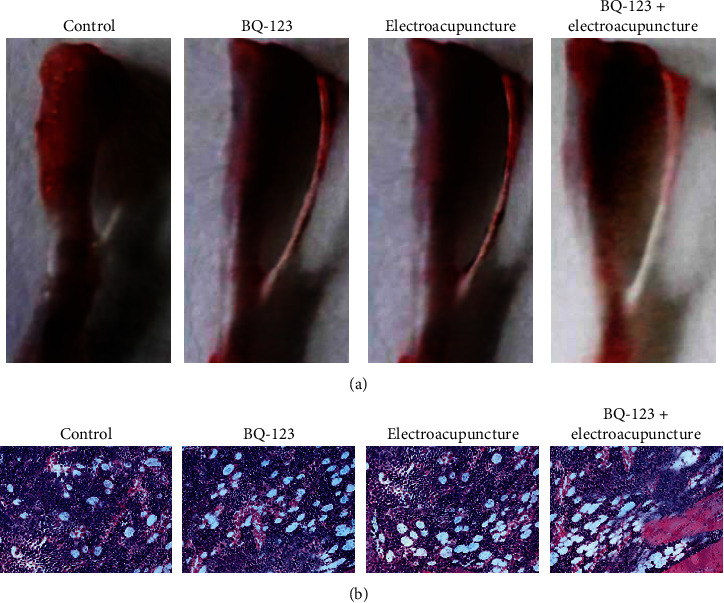
Anatomical and histopathological changes of the tibia in each group. (a) Anatomical pictures of the tibia. (b) HE dyed picture (×200).

Building after 18 days, the anatomy of the tibia was observed. As demonstrated in Figure 4(a), we found that in the control group, rats' tibia bone was destroyed, the bone surface feels rough, the heart stem epiphyseal end almost completely eroded, the tibia bone crisp is fragile, and you can see the empty sample change; BQ-123 part and the curative part contrasted with the control group belong to the moderate damage, BQ-123+ damage electric acupuncture group is lighter, and the bone is hard. The surface is complete and smooth to the touch. Building 18 days, after HE dyeing, we found that in the control group, rats tibial trabecular bone almost disappeared, the medullary cavity in tumor cell invasion, towards the outside bone, bone cortex damage is severe, and trabecular bone in BQ-123+ and the curative group was destroyed. There were a few tumor cells in the medullary cavity, and the overall damage was relatively mild, as shown in Figure 4(b).

### 3.5. Effects of BQ-123 Combined with Electroacupuncture on MWT and TWL in Rats

**Figure 5:**
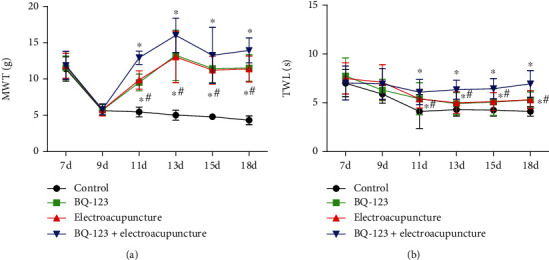
Effects of BQ-123 united with electricity on mechanical and thermal pain thresholds in rats. (a) Mechanical pain threshold (MWT) value. (b) Thermal pain threshold (TWL) value.

At 7-9 days, MWT and TWL values of each part were not statistically prominent (*P* > 0.05), 11-18 days, MWT and TWL values of BQ-123 and electroacupuncture part were prominently higher than those of the control part, the distinctions were statistically significant (*P* < 0.05), MWT and TWL values of BQ-123+ electroacupuncture part were prominently higher than those of BQ-123 and electroacupuncture part, and the distinctions were statistically significant (*P* < 0.05), as shown in Figures 5(a) and 5(b).

### 3.6. The Effect of BQ-123 Combined with Electricity on *α*-SMA, ETAR, and ETBR mRNA in Each Part

**Figure 6:**
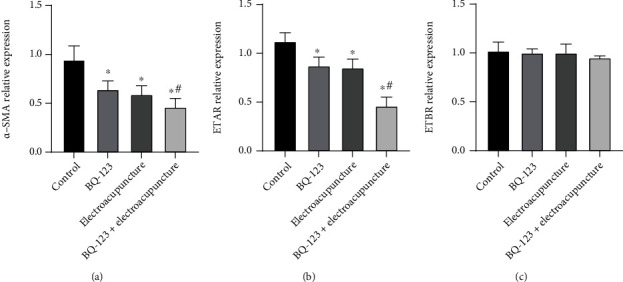
The effect of BQ-123 combined with electricity on *α*-SMA, ETAR, and ETBR mRNA in each group. (a) *α*-SMA mRNA expression. (b) Expression of ETAR mRNA. (c) Expression of ETBR mRNA.

The relative expression of *α*-SMA and ETAR mRNA in BQ-123 and electroacupuncture was significantly lower than in the control part, and the distinction was statistically significant (*P* < 0.05). The relative expression of *α*-SMA and ETAR mRNA in the BQ-123+ electroacupuncture part was prominently lower than that in BQ-123 and electroacupuncture group. The distinction was statistically significant (*P* < 0.05), as shown in Figures 6(a) and 6(b). The relative expression of ETBR mRNA in the four groups was not statistically significant (*P* > 0.05), as listed in Figure 6(c).

### 3.7. Effects of BQ-123 Combined with Electricity on PI3K/AKT Signaling Pathway

**Figure 7:**
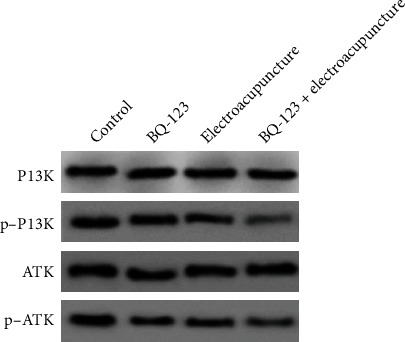
Effect of BQ-123 combined with electricity on PI3K/AKT signaling pathway.

Western blotting showed that there was no statistical significance in the expression of PI3K and AKT in each part (*P* > 0.05), the protein expressions of P-PI3K and P-Akt in the BQ-123 group and electroacupuncture part were prominently lower than those in the control group, and the distinction was statistically significant (*P* < 0.05). The protein expressions of P-PI3K and P-Akt in the BQ-123+ electroacupuncture part were significantly lower than those in the BQ-123part and electroacupuncture group. The distinction was statistically significant (*P* < 0.05), as shown in Figure 7.

## 4. Discussion

Bone cancer hurt caused by tumor metastasis to bone is one of the more common forms of pain in cancer patients. The pain caused by the destruction, squeezing, and invasion of tissues and cells caused by the rapid growth of malignant tumors is called cancer pain [13]. Relevant studies show that nearly half of patients with malignant tumors will suffer from pain, and 60%-84% of patients with terminal cancer have cancer hurt, which seriously impacts the living quality of patients [14]. Hyperalgesia means that the hurt response to stimulation is higher than normal, while hyperalgesia means that stimulation generates pain that should not cause pain [15]. Clinically, it is considered that the pathological mechanism leading to bone cancer pain is complex, mainly due to the gradual growth of cancer cells in the bone marrow cavity, leading to bone destruction and the continuous release of related cytokines, nerve cell remodeling and peripheral sensitization, and the continuous transmission of pain information to the central nerve and central axis sensitization [16]. Morphine belongs to opioids, which is more commonly used drugs in the treatment of osteocarcinoma pain, but with a greater incidence of the side effects of morphine, in addition to nausea, vomiting, constipation, and urinary retention, it also can produce addiction, and analgesic effect gradually declines, analgesic time obviously shortened, and resistance rate is as high as 57% [17], so it limits the clinical application of morphine. Therefore, it is crucial to study the pathological mechanism of osteocarcinoma pain and seek effective treatment drugs for clinical development.

Endothelin ET can be produced in various cells, released in the form of local hormones through autocrine and paracrine, and then combined with ETAR to exert its biological effects [18]. ET receptors are divided into ETAR, ETBR, and ETCR. ET receptors are divided into three types: ETAR, ETBR, and ETCR. ETAR has the highest affinity with ET-1 and the most potent force [19]. Relevant experiments have shown that inhibiting endothelin receptors can reduce myocardial cell damage caused by ischemia and hypoxia stimulation and have a protective effect on the myocardium [20]. At present, the ETAR antagonist is BQ-123, which has an essential protective effect on the brain, heart, lung, and other organs [21]. Acupuncture analgesia has a long history in China. Acupuncture can dredge the meridians, harmonize qi and blood, promote blood circulation, and relieve pain. Current clinical research has indicated that acupuncture has a good analgesic impact on inflammatory pain, neuralgia, and other acute and chronic pain [22]. Based on the above, this study mainly observed the effect of endothelin receptor A inhibitor BQ-123 combined with electroacupuncture on tibia cancer pain in rats and explored its related mechanism.

In this study, bone structure and behavior changes were contrasted between the sham part and the BCP group through tibial cancer modeling. The BCP group rats showed noticeable swelling of local tissues, bone trabecula loss, and bone cortex discontinuity. AS score was prominently higher than that of the sham part, and MWT were prominently lower than that of the sham part. The distribution of ETAR in the spinal cord dorsal horn was observed by the dual labeling method combined with laser confocal scanning. The results showed that most ETAR was colabeled with NeuN, and a small part was colabeled with GFAP and no colabeled with CD11b. Subsequent treatment experiments on BCP rats showed that BQ-123 combined with electroacupuncture was better than BQ-123 and electroacupuncture alone and effectively reduced the levels of *α*-SMA and ETAR mRNA.

P13K (phosphatidylinositol 3) is an oncogene and a critical signal transduction molecule in eukaryotic cells. Its downstream Akt belongs to the kinase of Ser and Thr proteins and is activated by phosphorylation of Threonine and serine on Akt [23]. Phosphatidylinositol 3-kinase-Serine-threonine protein kinase (PI3K-Akt) signal path can effectively regulate the classic signal cascade pathway of physiological and pathological mechanisms and participate in the processes of cell proliferation, migration, invasion, autophagy, and cycle arrest [24]. Phosphorylated inositol binds to Akt, the downstream target of P13K. The P13K/AKT signaling pathway is activated when AKT is phosphorylated through the serosa [25]. Studies have shown that PI3K/Akt signaling pathway refers to the protective mechanism of BQ-123 in the intervention of lung injury [26], but the role and mechanism of BQ-123 combined with electroacupuncture in the intervention of tibia cancer hurt in rats have not been determined yet. The consequence of this study manifested that the protein expressions of P-PI3K and P-Akt in BQ-123 group and electroacupuncture part were remarkably lower than those in the control part, and the protein expressions of P-PI3K and P-Akt in BQ-123+ electroacupuncture group were significantly lower than those in the BQ-123 group and electroacupuncture group. These results suggest that BQ-123 or electroacupuncture or BQ-123 combined with electroacupuncture may reduce the expression of *α*-SMA and ETAR by inhibiting the activation of PI3K-Akt signal path and effectively reducing the cancer pain of the tibia in rats. Relevant studies have shown that endogenous ET-1 can partially regulate P13K/Akt signaling pathway through its receptors, and the blockers of ETRA and ETRB, BQ123, and BQ788 can significantly inhibit the effect of hypoxia on increasing atrial P-Akt [27]. However, there are certain study limitations in the present study. The more clinical investigation should be performed to confirm our findings. In addition, further animal and cell studies need to be performed to confirm the findings of this study in our subsequent studies.

In conclusion, BQ-123 combined with electroacupuncture can relieve tibia cancer pain, mainly by inhibiting the activation of PI3K/Akt signal path and reducing the expressions of *α*-SMA and ETAR levels.

## Data Availability

All data generated or analysed during this study are included in this article.
